# Footwear in cricket: issues facing podiatrists treating fast bowlers

**DOI:** 10.1186/1757-1146-4-S1-P5

**Published:** 2011-05-20

**Authors:** Chris Bishop, Dominic Thewlis

**Affiliations:** 1 School of Health Science, University of South Australia, Adelaide

## Background

The choice of footwear and custom modification of footwear in fast bowling in cricket lacks biomechanical and clinical evidence. This demonstrates that despite professional advice being available, elite fast bowlers continue to wear shoes that may not be appropriate for them. The aim of this study was to investigate the biomechanical effects of three cricket shoes commonly used by fast bowlers and whether footwear modification could play a role in injury.

## Methods

Four male cricket fast bowlers were included in the study. A 20 camera VICON Mx system was used to collect three-dimensional kinematic data. A four segment marker set was used to track and model the lower limb. Four force platforms (Type 9287 BA, Kistler) were positioned at both back and front foot strike to capture kinetic data. Participants’ perceptions of footwear were measured using a VAS. Freidman two-way ANOVA with post hoc pairwise comparison was used to analyse the differences between shoes. Linear regression analysis was used to correlate predictive outcome measures.

## Results

The custom modified cross-trainer (ASICS 490tr) demonstrated the highest participant perception footwear score (mean 9/10 VAS). The conventional cricket shoes (ASICS 170no) demonstrated a significant reduction in front foot lateral shear force (*P* = 0.038) and a significant decrease in front knee joint external rotation moment (*P* = 0.022) relative to the custom modified shoe (ASICS 490TR). Exploratory regression analysis identified that front foot peak lateral shear force was significantly correlated (*R^2^* = 0.75, *P* < 0.001) to sagittal plane knee joint angle at initial contact. Sagittal plane knee joint angle at initial contact was also significantly correlated (*R^2^* = 0.73, *P* < 0.001) to front foot loading rate.

## Conclusions

The findings of this research demonstrate that custom modified cricket shoes increase lateral shear force and knee external rotation moment at the front leg. However, the relationship between these findings and injury remain unquantified. Further research must identify the role of footwear in the mechanism of lower limb injury in fast bowlers and what characteristics of footwear correlate to improved footwear VAS scores in regards to comfort, support and performance.

